# Sleeping Rooms

**Published:** 1861-02

**Authors:** 


**SLEEPING ROOMS.**—The air which passes out of the lungs is wholly innutritious. If re-breathed without any admixture of other air, it would induce instant suffocation. It contains a large amount of carbonic acid gas. This gas is condensed by cold, and falls to the floor; heat carries it to the ceiling; hence the practical fact, that in warm weather, those who sleep on the floor, breathe the purest air; while in very cold weather, the higher one sleeps above the floor, the better is the atmosphere. Hence, in a warm room, sleep as near the floor as possible; in a cold room, the higher the bed is, the better. A striking illustration of one branch of the statement is found in Dr. Hall's new book on SLEEP. When the jail-fever was raging in England, it was the custom to hand the food and water to the prisoners through a hole in the floor above them. A case is mentioned where the jailer and his wife died in one night, in consequence of the effluvia of the prisoners' cells below; while the prisoners themselves continued to live, showing conclusively the concentrated malignity of the air at the ceiling, as compared with that on the floor. The same principle has an illustration in the narration in the same pages, of the terrible incidents in connection with the "Black Hole of Calcutta," where it was speedily noticed that relief was given by sitting down on the floor. From these statements, it is clear, that it is better to have a fire in the fireplace in a close room in winter than to have no fire; and for two philosophical reasons—the fire rarefies the carbonic acid gas, and compels it to seek the ceiling; besides, it creates a draft up the chimney, thus causing cold air to come in more copiously through the crevices of the room; the inevitable effect of which is, a more copious supply of fresh air, and a more rapid change of air. Another incidental benefit from having a fire in the fireplace of a close room in winter is, that less bed-clothing is needed; hence the body is less smothered and sweltered; less oppressed by its own emanations, which are necessarily kept in more or less immediate contact with it, as the bed-clothing is heavier. When it is not convenient to build a fire in the fireplace, a good substitute is had in a large lamp, or jet of burning gas, brought into the fireplace by a flexible tube. These suggestions merit special reflection, as there seems to be a very prevalent opinion that cold air is necessarily pure, and that warm is a synonym of impurity.

